# The Fate of Microcystins in the Environment and Challenges for Monitoring

**DOI:** 10.3390/toxins6123354

**Published:** 2014-12-12

**Authors:** Justine R. Schmidt, Steven W. Wilhelm, Gregory L. Boyer

**Affiliations:** 1 Department of Chemistry, College of Environmental Science and Forestry, State University of New York, Syracuse, NY 13210, USA; E-Mail: glboyer@esf.edu; 2 Department of Microbiology, University of Tennessee, Knoxville, TN 37996-0845, USA; E-Mail: wilhelm@utk.edu

**Keywords:** microcystins, food web, microbial degradation, metabolism, glutathione metabolic pathway, toxicokinetics

## Abstract

Microcystins are secondary metabolites produced by cyanobacteria that act as hepatotoxins in higher organisms. These toxins can be altered through abiotic processes, such as photodegradation and adsorption, as well as through biological processes via metabolism and bacterial degradation. Some species of bacteria can degrade microcystins, and many other organisms metabolize microcystins into a series of conjugated products. There are toxicokinetic models used to examine microcystin uptake and elimination, which can be difficult to compare due to differences in compartmentalization and speciation. Metabolites of microcystins are formed as a detoxification mechanism, and little is known about how quickly these metabolites are formed. In summary, microcystins can undergo abiotic and biotic processes that alter the toxicity and structure of the microcystin molecule. The environmental impact and toxicity of these alterations and the metabolism of microcystins remains uncertain, making it difficult to establish guidelines for human health. Here, we present the current state of knowledge regarding the alterations microcystins can undergo in the environment.

## 1. Introduction

Cyanobacteria (blue-green algae) are photosynthetic organisms found in both marine and freshwater environments. These organisms can be benthic or pelagic [1]. Selected strains of cyanobacteria are capable of fixing atmospheric nitrogen, giving them a competitive edge over other algal species [1,2]. Cyanobacteria can also avoid predation by forming colonies and elongated shapes, making predatory grazing difficult [3]. Many species of cyanobacteria produce secondary metabolites, some of which are toxic to higher organisms [4]. Toxic metabolites are classified into four categories: hepatotoxins, dermatoxins, neurotoxins and cytotoxins [5,6,7]. The occurrence of algal blooms has increased over time [8], and anthropogenic input of phosphorus and nitrogen into natural water bodies are contributors to this increase in toxic algal blooms [9]. The risk to human health due to the increasing presence of these toxins is of concern [9,10,11].

**Figure 1:**
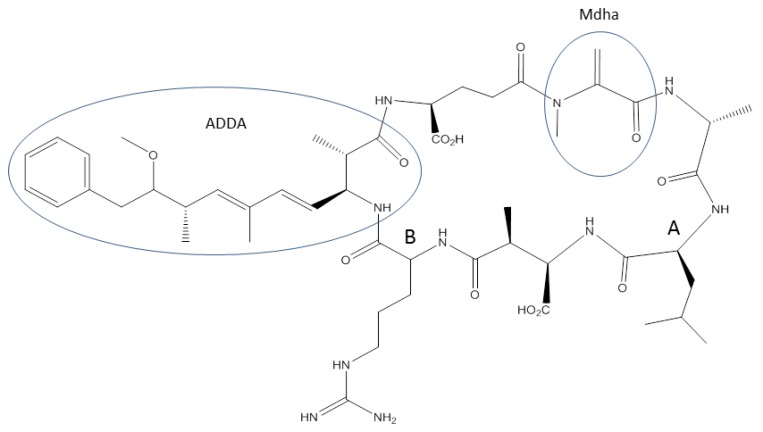
The structure of microcystin-LR. The 3-amino-9-methyoxy-2,6,8-trimethyl-10-phenyl-4,6-decadienoic (ADDA) and methyl-dehydroalanine (Mdha) groups are important in binding of microcystin-LR to the protein phosphatase enzyme. The microcystin ring system contains two variable sites (A and B), which can contain variable amino acids. This forms the basis of microcystin nomenclature. Microcystin-LR contains leucine at site A and arginine at site B, microcystin-RR contains arginine at both sites, *etc*.

**Figure 2:**
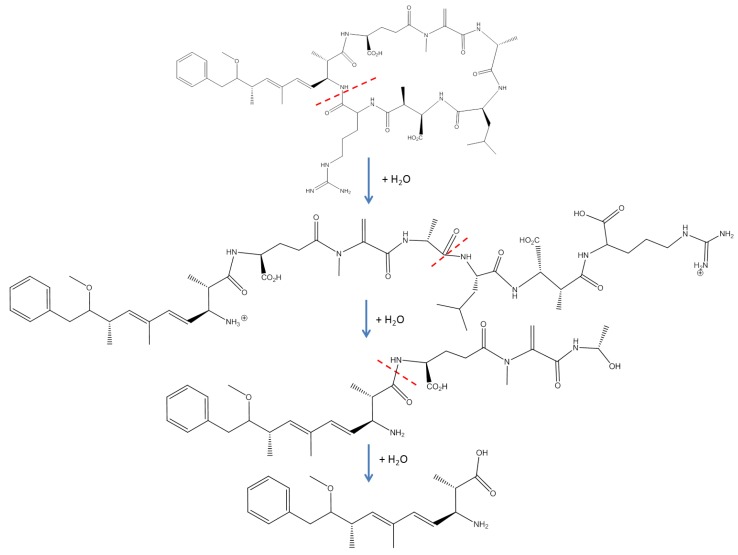
Degradation pathway of microcystin-LR by bacteria. The first step is the cleavage of the peptide ring at the ADDA-arginine bond, followed by subsequent degradation of the linear microcystin-LR product to yield a tetrapeptide intermediate and the ADDA moiety. Sites of enzymatic cleavage are indicated with dashed lines (derived from mechanism proposed by [60]).

Microcystins are a group of over 90 hepatotoxins produced by cyanobacteria, of which microcystin-LR (MC-LR) is the most common [7,12,13] (Figure 1). Their toxicities resulted from the inhibition of protein phosphatases and disrupted formation of the cytoskeleton [7]. They also promote oxidative stress in liver tissues [2]. Microcystins can be introduced to tissues of organisms through the diet or by ingestion of contaminated water [2,14,15]. The log of the octanol/water distribution ratio, (*i.e.*, logD_ow_ in reference [16]) of microcystin-LR is approximately −1 at pH 7 (estimated from Figure 2 in reference [16]), indicating that microcystins are readily water-soluble and not likely to passively diffuse into tissues from the surrounding water. Instead, microcystins accumulate in the liver via the bile acid transport system [7,14,17,18]. The bile acid transport system is comprised of proteins that actively transport peptides and biliary acids into hepatocytes [19]. The methyl-dehydroalanine (Mdha) and 3-amino-9-methyoxy-2,6,8-trimethyl-10-phenyl-4,6-decadienoic acid (ADDA) groups are integral to binding of microcystins to protein phosphatases in organisms (Figure 1) [20].

The toxicity of microcystins varies according to the combination of amino acids at the two variable positions on the peptide ring (Figure 1) [12,21]. The oral LD_50_ for MC-LR in rats and mice is 5 mg/kg of body weight [22,23]. For comparison, the oral LD_50_ for cyanide is 3 mg/kg of body weight [24]. The oral LD_50_ for microcystin-RR (MC-RR), a microcystin congener with two arginine groups attached to the peptide ring, is ten-fold higher than that of MC-LR [23]. The LD_50_ for MC-RR and MC-LR administered intraperitoneally in mice are 235.4 and 43 µg/kg body weight, respectively [15]. MC-RR is more polar than MC-LR and is not as easily transported via the bile acid transport system as MC-LR, hence the observed difference in toxicity [12]. Microcystin-LA (MC-LA), which has leucine and alanine at the two variable positions on the peptide ring, has an intraperitoneal LD_50_ identical to that of MC-LR [25,26,27,28]. Different microcystin congeners vary in their response to the protein phosphatase inhibition assay (PPIA). For example, the half maximal inhibitory concentrations (IC_50_) ranged from below 0.06 µg/mL for [D-Asp3, Z-Dhb7] MC-LR to greater than 10 µg/mL for MC-RR in the PPIA [29]. MC-LR is an environmental health concern due to its widespread presence in freshwater bodies worldwide.

Human exposure to microcystins is recognized as a global health issue [9,10,11]. Incidents of human sickening [30] and death [31] due to microcystin exposure have been documented. Microcystin exposure may promote liver tumors [32] and has been linked to liver cancer in humans [33]. Chronic exposure to low levels of microcystins can promote the growth of tumors in the liver and other organs [23]. Human health guidelines have been made for the unaltered toxins in water and in tissues [23,34,35], but updating these regulations to include correction factors for environmental alterations is a challenging problem. The environmental fate of microcystins affects its impact on human health.

### 1.1. Abiotic Transformations

Dilution is a major process by which microcystin toxicity is reduced in natural waters [36,37]. Microcystins are released into the surrounding environment after a bloom senesces or cells are ruptured. Dilution of microcystins occurs when they are introduced to a large volume of water, such as that of a lake. However, dilution may not always reduce microcystin abundance below the critical point for organisms, as some deep oligotrophic lakes are also subject to toxic algal blooms [38]. Breakdown of microcystins due to high temperatures has also been cited as a means for detoxification [23,39]. However, thermal decomposition is usually partnered under laboratory conditions with low pH to increase the degradation rate, as microcystins are stable at high temperatures and can withstand boiling [23,36].

Microcystins are adsorbed onto suspended particulate matter (SPM) in aquatic systems [40,41]. Adsorption by SPM is an important mechanism of removal for contaminants in both freshwater and marine environments [41,42]. This process limits exposure of fish and other biota to free microcystins by binding to particles and may decrease the risk of microcystin food web transfer [41,43]. The extent to which microcystin is adsorbed by SPM is a function of the pH [16].

The microcystin molecule contains two carboxyl groups and one amino group not built into the peptide ring, which are able to be ionized (Figure 1) [44]. However, the very low pH necessary to ionize these carboxylic acid groups (approximately pH 2.1) is rarely seen in natural systems [16]. The pH of a water body increases during a productive algal bloom and can exceed pH 8 due to the consumption of dissolved carbon dioxide [45,46]. The adsorption of microcystins onto SPM is dependent on the hydrophilicity of the microcystin congeners at pH ranges found in natural waters (e.g., pH 6–9).

Despite the hydrophilic nature of microcystins in natural systems, microcystins are adsorbed onto sediments. Adsorption to sediments was stronger with more hydrophilic microcystins, such as microcystin-RR (microcystin-arginine and arginine, Figure 1), than with less hydrophilic congeners [37]. The affinity of microcystin-RR for sediments is dependent on the different mechanisms for microcystin adsorption onto particles. Adsorption can begin with hydrophilic groups on the microcystin structure interacting with sediments [37]. The most hydrophobic part of the microcystin molecule, the ADDA moiety (Figure 1), may not strongly interact with sediments. Alternatively, naturally occurring clays can be used to adsorb microcystins via interactions with the ADDA moiety, making hydrophobic binding the dominant process over hydrophilic interactions in these cases [40]. The precise mechanism for binding of microcystins to sediments is uncertain.

Microcystins are also subject to transformation from exposure to sunlight in the presence of photosensitizers [47,48]. Isomerization of the conjugated ADDA side chain from the “E” to “Z” configuration of the microcystin molecule can occur [49,50,51]. This process requires low concentrations of photosensitizers, such as humic acids or pigments; microcystins cannot be transformed by sunlight alone [47,48,52]. The rate of microcystin transformation by light is dependent on several factors, including pH, concentration of humic acids and wavelength of light. In natural systems, large concentrations of humic acids may shield microcystins from being altered by sunlight, which explains the persistence of microcystins in water samples exposed to lower energy ultraviolet light and sunlight [43,51,53].

Breakdown of microcystins can also occur through photodegradation [36,43,49]. MC-LR has been degraded at the conjugated diene and aromatic ring of ADDA and at the double bond at the Mdha position using titanium oxide in the presence of ultraviolet light or coupled hydrogen peroxide and an iron-yttrium complex in the presence of visible light [41,53,54,55,56,57]. Complete degradation of microcystins was observed within five days using 365-nm light and within only 1 h using 254-nm light [43]. The majority of MC-LR (79%) was degraded when exposed to the full spectrum of ultraviolet light for 22 days [52]. The half-life of MC-LR in a natural system is estimated at 90–120 days per meter of water depth [48]. A decrease in the pH of a water body will increase the rate of microcystin photolysis [51], but the pH of the surrounding water increases during a productive algal bloom. Thus, the transformation of microcystins is only significant in shallow water bodies due to the availability of sunlight [48]. High pH and high concentrations of humic acids may prevent exposure of organisms to critical levels of microcystins in natural systems.

### 1.2. Biological Processes

#### 1.2.1. Microbial Degradation

Microbial degradation of microcystins has been confirmed as a detoxification mechanism in selected strains of fungi [58,59] and bacteria [60,61,62]. In selected strains of bacteria, three enzymes collectively referred to as microcystinase operate in a sequential pathway to degrade MC-LR [61]. The first enzyme linearizes MC-LR through the cleavage of the peptide ring at the ADDA-arginine bond. The second enzyme cleaves this linear intermediate at the alanine-leucine bond, yielding a peptide intermediate of ADDA-Glu-Mdha-Ala (Figure 2) [60,61,62]. The final enzyme degrades the products formed by the first two enzymes and liberates ADDA from the tetrapeptide intermediate (Figure 2). ADDA is non-toxic up to 10 mg/kg [63].

Degradation of MC-LR in a laboratory study began almost immediately once the bacterium *Sphingopyxis* sp. C-1 was introduced to MC-LR [64]. After 24 h, 25% of the MC-LR was degraded. The levels of MC-LR were at or below the World Health Organization “safe” level of 1 µg/L in drinking water by the eighth day of treatment with *Sphingopyxis* sp. C-1 [27]. Bacterial degradation contributes to the detoxification of microcystins in a laboratory setting at neutral pH.

Bacteria that thrive in water with productive algal blooms need to be tolerant of alkaline conditions [46]. The maximum degradation rate of MC-LR was observed between pH 6.5 and 8.5 for *Sphingopyxis* sp. C-1, an alkaline-tolerant bacterium, even though the optimal pH for growth of the bacterium was at approximately pH 7.0 [65]. Immediately after MC-LR was added to lake water containing native microbes and a culture of the microcystin-producing cyanobacterium, *Microcystis aeruginosa*, degradation of MC-LR was initiated, and the diversity of microbes increased [66]. The microbial community surrounding a cyanobacterial bloom in natural environments may vary by location [67]. *Oscillatoriales*, *Chroococcales* and *Nostocales* were dominant in three lakes with toxic blooms over two continents, indicating that the bacterial community in a toxic bloom at a phylogenetic level is conserved across lakes [67]. The phyla of the bacterial communities in these lakes were different; genetic signatures for nitrogen uptake in Lake Erie and Grand Lake St. Marys in North America were dominated by cyanobacteria, whereas the bacterial community in Lake Tai, China, was dominated by Proteobacteria [67]. In Lake Tai, certain species of eubacteria may be closely linked to blooms and could potentially use toxins produced by the bloom event as a carbon source [68].

Although bacteria are numerous and very diverse, not all bacterial strains are able to break down microcystins. Selected strains of *Sphingomonas sp*. and *Sphingopyxis sp*. are capable of microcystin degradation [61,64,69,70,71]. Novel bacterial species that degrade microcystins have also been identified in water [72,73]. Lahti *et al.* (1998) characterized 17 strains of bacteria that degrade microcystins [74]. Berg (2009) identified 460 strains of bacteria present in water bodies with frequent cyanobacterial blooms, in which *Sphingomonas* was the dominant species [75]. Microcystins could accumulate and remain in the water column if the particular bacterial strains that degrade microcystins are not present during a toxic bloom [69,74]. Bacterial species capable of degrading microcystins have also been identified in soils [28].

#### 1.2.2. Metabolism and Conjugation

The ADDA group on MC-LR is important for the binding of the toxin to its target enzyme, protein phosphatases 1 and 2A [76]. The Mdha group in microcystins can subsequently covalently bind to a cysteine in a protein phosphatase enzyme [20]. Microcystins permanently block the active site and destroy the functionality of the protein phosphatase enzyme. Organisms subject to microcystin exposure have developed detoxification mechanisms to resist microcystin intoxication. Animal metabolism utilizes two classes of enzymes to eliminate xenobiotics [77,78]. The primary phase I enzymes are usually the cytochrome P-450 enzymes, which catalyze the addition of oxygen-containing groups to toxins through oxidation-reduction reactions. Products of phase I enzymes are often reactive and could negatively affect the cell if not metabolized further by phase II enzymes. These products are then conjugated to glucuronic acid, sulfates or peptides to prevent cellular damage [78].

**Figure 3:**
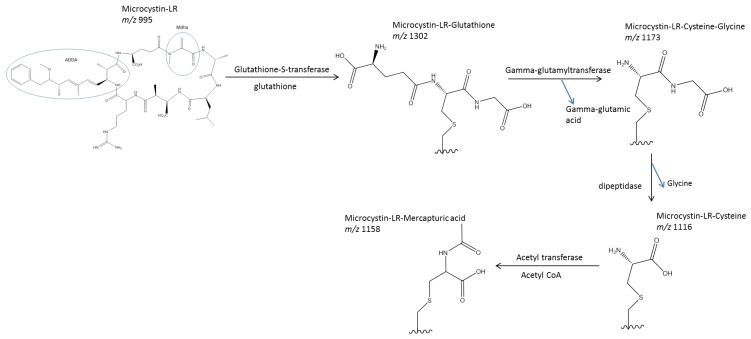
Glutathione metabolic pathway for microcystin-LR. Metabolic alteration using the glutathione pathway occurs at the double bond of the methyl-dehydroalanine (Mdha) position on the peptide ring. The 3-amino-9-methyoxy-2,6,8-trimethyl-10-phenyl-4,6-decadienoic acid (ADDA) group is responsible for non-covalent binding to protein phosphatase. After the first enzymatic step, microcystin-LR is represented by a rectangle for each step, with the chemical structure of the metabolically altered groups drawn out. Glutathione is attached to the Mdha group of microcystin-LR through glutathione-S-transferase, forming the microcystin-LR-glutathione conjugate. The gamma-glutamic acid group is removed by gamma-glutamyltransferase, yielding the microcystin-LR-cysteine-glycine conjugate. The glycine group of the microcystin-LR-cysteine-glycine conjugate is removed by dipeptidase, forming the microcystin-LR-cysteine conjugate. This microcystin-LR-cysteine conjugate is oxidized through acetyl transferase, forming the microcystin-LR-mercapturic acid conjugate.

Glutathione (GSH) is a common peptide used in phase II biotransformations (Figure 3) [78]. Formation of the glutathione conjugate by the phase II enzyme, glutathione-*S*-transferase (GST), is one of the most common types of xenobiotic modification. This reaction occurs between a nucleophilic center on the toxin and the sulfhydryl group of the reduced glutathione (Figure 3) [79]. In microcystins (Figure 1), this nucleophilic center is provided by the Mdha group. Conjugation of GSH to a xenobiotic increases its water-solubility so that the toxin can be eliminated. Glutathione-conjugated xenobiotics can be eliminated via bile [80,81], although this is rare [78].

GSH-conjugation is often the first in a series of metabolic alterations to a xenobiotic, ultimately producing an *N*-acetyl-cysteine (mercapturic acid) conjugate that is quickly excreted by the organism (Figure 3) [78]. The γ-glutamic acid group of the GSH molecule is enzymatically cleaved by gamma glutamyl transferase, forming the γ-glutamylcysteine intermediate (Figure 3) [78,82]. The glycine of this γ-glutamylcysteine intermediate is then cleaved by a dipeptidase to yield the cysteine-conjugated product, which is subsequently oxidized to form the mercapturic acid metabolite (Figure 3) [78]. This mercapturic acid derivative is easily excreted in urine, although there is potential for the other conjugates in the metabolic pathway to be removed from cells for excretion [78]. A glutathione-*S*-pump is an active transport protein, which moves GSH-conjugated toxins out of cells and could potentially transport microcystins, although no experiments with this pump transporting MC-LR-GSH have been conducted [83,84,85].

The processing of MC-LR either occurs via irreversible covalent binding to proteins or biotransformation through the glutathione pathway into intermediate products (Figure 3) [86,87,88,89,90]. It is assumed that covalently-bound microcystins are not a major vector for microcystin exposure [91]. However, bound microcystins have the potential to be cleaved enzymatically, releasing peptide fragments, which can be liberated from tissues [92,93]. These microcystins with their attached peptide fragments still possess some degree of protein phosphatase inhibition by PPIA [92]. This represents an additional pool of potentially toxic microcystin analogs in the cell. These fragments could be released over a period of time as enzymatic cleavage occurs, creating another source of microcystins for potential exposure to organisms.

Conjugation of microcystins to GSH or other proteins is a major portion of the microcystin pool in natural systems [91]. Proteins that microcystins can bind to may be within the toxic bloom itself rather than in the cells of an exposed organism. Microcystins can bind to cysteine residues in several proteins in *Microcystis* through a Michael addition in the same manner as they conjugate to GSH [94]. This binding occurs more rapidly when *Microcystis* cells are under oxidative stress, which is similar to the intercellular environment of organisms in the presence of microcystins [94]. These conjugated microcystins may not be detected using traditional analytical methods, such as LC-MS or ELISA, due to the many possible proteins that can react with microcystins [91,95].

Studies on the zooplankton, *Daphnia magna*, the freshwater mussel, *Dreissena polymorpha*, and the macrophyte, *Ceratophyllum demersum*, suggest that the first step in the biotransformation pathway is the attachment of glutathione onto the Mdha residue of MC-LR [87,89]. This pathway is conserved between these highly diverse organisms. The glutathione conjugate is enzymatically converted into the cysteine conjugate through a cysteine-glycine intermediate. The final conjugate formed in this biochemical pathway is the mercapturic acid conjugate (Figure 3). Free (unconjugated) MC-LR can be eliminated via urine and feces in mice [96] and bivalves [97,98]. Conjugate intermediates have also been detected in feces [18] and bile [78,99,100,101] of rats. MC-LR-GSH was concentrated in feces in rats, and MC-LR-Cys was concentrated in kidneys, indicative of excretion [18]. Both the glutathione-conjugated MC-LR (MC-LR-GSH) and cysteine-conjugated microcystin-LR (MC-LR-Cys) conjugates have been identified in mice [85,102], rats [103], fish [104,105,106,107,108], shrimp [106] and snails [106]. The MC-LR-GSH conjugate has also been identified in humans [109].

These biological detoxification pathways are important in determining the toxicity of microcystins in an ecosystem. Biotransformation removes microcystins from the organism and releases them back into the environment. MC-LR-GSH is three- to ten-times less toxic to mammals than free MC-LR [18,110,111]. The effects of the glutathione and cysteine conjugates of MC-LR are the same as those noted from exposure to free toxin, such as hepatocyte damage [18,110,112]. Non-covalently bound MC-LR in zooplankton was readily taken up by the intestines of the planktivorous sunfish, *Lepomis gibbosus* [113]. More than 80% of free, non-covalently bound microcystin in the zooplankton *Bosmina* fed to *Lepomis gibbosus* was directly transferred to the sunfish. Free and conjugated microcystin-LR (MC-LR-GSH, MC-LR-Cys, *etc*.) can travel up the aquatic food web [113]. MC-LR was more efficiently transferred between organisms using zooplankton as a vector than by direct transfer of the toxin to *Lepomis gibbosus* through exposure to contaminated water. However, these results are complicated by the use of the enzyme-linked immunosorbent assay (ELISA) to determine microcystin concentrations in tissues. Matrix effects from tissue extracts complicate the use of ELISA to determine microcystin concentrations below 5.9 µg/kg dry weight [114]. MC-LR and its conjugates could transfer to organisms after excretion and be released into the environment [113]. *Daphnia* has been implicated as a potential vector for food web transfer of microcystins due to its consumption of toxic cyanobacteria as a food source [115].

The World Health Organization (WHO) has instituted a daily tolerable intake for lifetime human exposure of MC-LR of 0.04 µg/kg of body weight/day limit from food, based on studies in rats [22,23,27]. This has been expanded upon to create seasonal daily exposure tolerable intake of MC-LR in food of 300 µg/kg of food for adults and 40 µg/kg of food for children [34]. An acceptable daily limit of 39 µg/kg of fish for adults (aged 17 and above) and 24 µg/kg of fish for children (aged 2–16) was derived based on the No Observed Adverse Effect Level (NOAEL) of 40 µg/kg/day [35]. However, these regulations are based on the toxicity of the parent toxin, not the metabolic products.

It is currently uncertain if microcystins bioaccumulate in organisms or if they biomagnify in food webs. Bioaccumulation is defined as the concentration of a compound in a specimen being higher than that of its surrounding environment, including water and food, and biomagnification is defined as the concentration of a compound being increased from one trophic level to another in a food web [116,117]. Microcystins can bioaccumulate in the liver, muscle and viscera of the omnivorous fish, *Tilapia rendalli* [118]. No microcystins were detected in the phytoplankton samples collected from the lagoon field site, but microcystins were detected in fish tissues. Similarly, microcystins were detected in the tissues of the freshwater mussel, *Anodonta grandis simpsoniana*, despite microcystin levels in the surrounding water being below detection [119]. In both cases, the levels detected in tissues were above the WHO guideline for human exposure to MC-LR [118,119]. Differences in microcystin content by trophic level have been observed. MC-LR, MC-RR and microcystin-YR were detected in fish from Lake Tai, China, with the highest levels found in omnivorous fish, followed by phytoplanktivorous fish and carnivorous fish [120]. This observed accumulation of microcystins in tissue after toxin levels were reduced below detection in water could reflect a retention of toxins in the tissue after toxins had disappeared from the water column. This could also reflect bound microcystins being gradually released within the tissue in the form of microcystin-containing peptide fragments [92].

Bioaccumulation of microcystins into fish tissues is also complicated by the high variability of toxin concentration in natural systems [95,121]. Out of five species of fish taken from Grand Lake St. Marys in Ohio, USA, only two had detectable levels of MC-LR in muscle tissues [95]. This implicates a species-dependent mechanism of MC-LR uptake into fish. However, fish of the same species had a range of toxin values from non-detection to as high as 70 µg/kg, which is over three orders of magnitude above the World Health Organization guideline for tissue [95]. There is clearly variation in toxin uptake between individual fish of the same species, as well, which can be a major complicating factor when addressing human health guidelines. Even if a safe toxin threshold value is established for consumption of fish tissues of a particular species, it is impossible to determine if any given fish is below this threshold or orders of magnitude above it.

Some ecosystems have organisms with consistently high levels of microcystins in their tissues. Amrani *et al.* (2014) reported that common carp muscle was over the 0.04 µg/kg of the body weight/day guideline value from food over a one-year period of monitoring carp tissue using PPIA [122]. Both PPIA and ELISA are subject to interferences from tissue matrices [95]. Peng *et al.* (2010) reported that aquatic organisms from three lakes in China were as high as 148 times the WHO safe guideline value of 0.04 µg/kg body weight/day [123]. Over half of the fish tissues from Lake Tai in China contained levels of microcystins over the WHO guideline using tandem mass spectrometry [124]. Organisms from these Chinese lakes were considered potentially harmful to humans if consumed.

There is also evidence for biodilution, rather than biomagnification, of microcystins in aquatic food webs. Biodilution is defined as the concentration of a parent analyte (not metabolically altered) decreasing in organisms as the trophic level increases [125,126]. Accumulation of microcystins in tissues is dependent on the species [95,127]. Biodilution is dominant over biomagnification in most aquatic species, with the exceptions being zooplankton and planktivorous fish [113,127]. The entire body of the organism is generally used for toxin analysis when examining zooplankton and planktivorous fish tissues, which could account for the observed biomagnification in these species. 

No evidence of bioaccumulation was found in shrimp, mussels, frogs or fish in a eutrophic lake [128]. Rather, the tissue microcystin concentration was at or below that of the phytoplankton in the lake water. The concentration of microcystins in tissues in the literature may be skewed due to comparisons between individual organs and whole organisms, such as zooplankton, giving a false impression of biomagnification [129].

## 2. Degree of Resistance to Microcystin Toxicity

Organisms can avoid microcystin intoxication through adaptations in their diet. Selected species of fish and zooplankton will preferentially graze on non-toxic phytoplankton when both a toxic and non-toxic strain are present [46]. By avoiding consumption of toxic algal strains, organisms reduce their risk of microcystin exposure. In environments where this selectivity is not possible, such as water bodies with very large toxic blooms, organisms can adapt by developing a tolerance to microcystin toxicity [118,130,131].

The degree to which an organism is resistant to microcystin toxicity is variable between species. Bivalves, such as *Dreissena polymorpha* and *Mytilus galloprovincialis*, can acclimate to microcystin exposure and accumulate measurable concentrations of microcystins in their tissues without mortality [86,97,132,133,134,135,136,137]. Some species of *Daphnia* also show variable sensitivity to microcystin exposure [138].

### 2.1. Terrestrial Organisms vs. Aquatic Organisms

There is a difference in the physiological response to microcystin toxicity between terrestrial and aquatic organisms. Oral LD_50_ measurements for microcystins are based upon rat and mouse bioassays [22,23]. Mouse mortalities from microcystin exposure resulted from hemorrhaging of hepatocytes [22]. Fish in laboratory studies dosed with microcystins can remain alive longer and at higher levels of toxin than mice [139,140]. Rainbow trout did not experience mortality when exposed to not only higher doses of microcystins than mice, but also when exposed to a longer duration of microcystin exposure [141]. There were no signs of hepatocyte hemorrhage in rainbow trout as observed in mice [141,142]. Mice that overexpressed a transcription factor associated with preventing cellular damage due to oxidative stress experienced less liver damage than mice without this overexpression when exposed to microcystin-LA [142]. Mice may thus have a mechanism for the prevention of hepatocyte damage due to microcystin exposure that is induced by oxidative stress [142].

Rats showed signs of liver hemorrhage rather than kidney damage, as seen in selected species of fish [141,143]. Hepatocyte hemorrhage was not observed in rainbow trout exposed to MC-LR, differing from studies in rats and other fish, indicating that rainbow trout may be more tolerant of microcystin exposure than other organisms [141]. Fish may have a mechanism through urinary elimination of microcystins from the liver that differs from that of rats that decreases hepatocyte damage due to microcystin exposure. Selected species of fish in environments prone to toxic blooms must have a mechanism for quick, efficient removal of microcystins to prevent mortality. 

Aquatic organisms exposed to microcystins may also develop a resistance to intoxication and can adapt to toxic environments. Nymphs of the burrowing mayfly, *Hexagenia*, were able to withstand MC-LR levels up to 10 µg/L MC-LR, a concentration recorded during a very toxic bloom in a highly eutrophic lake in North America [144,145]. Only 10% of hatchlings at the WHO guideline of 1 µg/L for drinking water died during one week of exposure, and only 20% died after one week of 10 µg/L MC-LR exposure. Large nymphs experienced less mortality than smaller nymphs, though this could be due to a difference in the surface-to-volume ratio between organisms. Alternately, larger nymphs may have more developed metabolic pathways to detoxify MC-LR than younger ones. Since *Hexagenia* were taken from water bodies with histories of toxic algal blooms, these particular organisms may have developed a resistance to MC-LR due to prior exposure to the toxin, enabling them to withstand exposure to high concentrations [145].

### 2.2. Bivalves

Studies on freshwater bivalves have shown measureable concentrations of microcystins in the viscera [86,97,132,133,134,135,136]. Little to no animal mortalities were reported in these studies, indicating that these organisms may have a higher resistance to microcystins than other organisms [136]. In the freshwater mussel, *Mytilus galloprovincialis*, an increase in GST activity in the gut and a marked decrease in GST activity in the labial palps were observed when exposed to MC-LR. The labial palps may act as a control on the concentration of microcystin taken into the gut of the bivalve, as secretion of mucus by these organs would restrict intake of toxic *Microcystis* [136].

Bivalves may also resist microcystin toxicity through expulsion of *Microcystis* in the form of pseudofeces [135,136]. *Dreissena polymorpha* is indiscriminate in the uptake of cyanobacterial strains as a food resource, regardless of toxicity [134]. However, *D. polymorpha* will produce pseudofeces in the presence of toxic cyanobacteria [98]. In the laboratory, *D. polymorpha* was exposed to both toxic *Microcystis aeruginosa* and the non-toxic diatom, *Asterionella formosa* [135]. Pseudofeces produced by these zebra mussels contained mostly *Microcystis*, not the non-toxic diatom. Thus, zebra mussels were selectivity avoiding *Microcystis* [135]. A subsequent experiment that would provide more data on whether bivalves are selectively avoiding toxic *Microcystis* is to feed both toxic and non-toxic *Microcystis* to these bivalves and observe which food is eaten and excreted. This way, differences in the morphology and size of the two foods can be better controlled. Bivalves in general may have a greater resistance to toxicity than other organisms [137]. An increase in the multidrug resistance protein activity was immediately observed when *Dreissena polymorpha* was exposed to MC-LR, as well as other xenobiotics [137]. Bivalves may be better equipped than other aquatic organisms for resisting toxicity [137].

### 2.3. Fish

Development of microcystin tolerance has been documented in fish. The physiological response of fish to microcystins can be attributed to several factors, including the length of time of exposure and dosage [139,141]. The age and size of the fish may also be a factor, as the concentration of microcystins in muscle tissue decreased with the length of the fish [146]. Microcystin levels were over 500 µg/g dry weight from algal samples from Grand Lake St. Marys in Ohio, USA, in 2010 [95]. Fish collected in 2011 and 2012 had detectable levels of MC-LR in their muscle tissues, whereas water samples collected during 2011 and 2012 did not show microcystin concentrations above the detection limit by LC-MS(/MS) [95]. The persistence of MC-LR in fish tissues when no microcystins were detected in the lake water indicates that these fish may accumulate the toxins and may have a mechanism protecting them from experiencing mortality from microcystin exposure.

The goldfish, *Carassius auratus* L., accumulated MC-LR in the liver after intraperitoneal injection [140]. The concentration of MC-LR in liver cells increased between 0 and 48 h of microcystin exposure [140]. The ionic homeostasis of all test organisms remained unaffected by MC-LR addition, despite high levels of MC-LR. However, a rapid decrease in liver MC-LR concentration and recovery of damaged hepatocytes were observed after 48 h. A similar trend was observed in the common carp, *Cyprinus carpio* L. [139]. Damage to kidneys observed both in goldfish and in carp indicated that MC-LR was passing through kidney cells to be excreted [139,140]. MC-LR and the MC-LR-Cys conjugate were found in the kidney tissues of fish from Chinese lakes, implicating the kidney as an excretory route for MC-LR and its metabolites [106,147].

### 2.4. Zooplankton

Zooplankton are an important part of the food web in natural systems and exhibit species-dependent degrees of resistance to microcystin toxicity. The rate of zooplankton grazing on cyanobacteria is not dependent on microcystin concentration alone. Cyanobacteria can produce secondary metabolites, such as protease inhibitors and other anti-feedants, which reduce grazing by zooplankton [2,148]. Toxic compounds, such as microcystins, have traditionally been correlated to reduce feeding by zooplankton [149,150,151,152,153]. Grazing on a toxic bloom by *Daphnia sp*. was not inhibited by toxins, despite poisoning of *Daphnia* due to the ingestion of toxic cells indicated by abnormal swimming behavior [154,155]. Cyanobacteria have also been cited as a poor food source for zooplankton due to low fatty acid and overall nutritional content, which could reduce the rate of feeding [149,156,157,158]. It has also been suggested that *Daphnia* grazing on toxic *Microcystis* strains in an environment where non-toxic *Microcystis* is also growing helps protect the non-toxic strains, as the *Daphnia* feeding rate slows in the presence of the toxin-producing *Microcystis* [159].

MC-LR is a strong inhibitor of protein phosphatases in zooplankton [160]. The copepods, *Acartia bifilosa* and *Eurytemora affinis*, experienced a decrease in survival when exposed to MC-LR in filtered water culture medium at concentrations found in natural water bodies. These copepods were more sensitive to MC-LR than daphnids [161,162,163]. When toxic blooms are present, copepods have the ability to selectively feed and exclude toxic algae [164]. The copepod *Diaptomus birgei* excluded toxic cyanobacterial strains and also nontoxic cyanobacteria that had a similar morphology to toxin-producing species [151,152,164]. This selectivity provides a mechanism for copepods to prevent microcystin intoxication.

Cladocerans, such as *Daphnia*, are relatively non-selective feeders and cannot exclude toxic cyanobacterial strains from the diet [3]. *Daphnia* of different species do not have equivalent responses to microcystin exposure [138,160,165]. Of four *Daphnia* species examined, *Daphnia magna* was the least resistant to toxicity and experienced feeding inhibition in the presence of toxic *Microcystis* after 1 h of exposure [165]. *D. galeata* did not show any sign of feeding inhibition over a 1-h period under the same feeding conditions as *D. magna*, and the feeding rate of *D. pulex* was more inhibited than that of *D. pulicaria* [160,165]. Other species demonstrated varying degrees of growth inhibition. Both inhibition of feeding and growth indicate that *Daphnia* are negatively affected by the presence of toxic *Microcystis*. Varying sensitivity among species of *Daphnia* is partly due to physiological differences [161].

The age of *Daphnia* also makes a difference in response to microcystins. Younger *Daphnia* may be better adapted to survival in an environment with microcystins, as increased glutathione-S-transferase (GST) activity in response to microcystin toxicity decreased with age [131,166]. The extent of damage to organisms by microcystins varies between *Daphnia* clones, as well as by age, and it can be difficult to assess the organismal effect of microcystin exposure.

Daphnids that had prior microcystin exposure experienced less mortality when exposed to microcystins than species that did not [130,131]. *Daphnia galeata* hatched from resting eggs formed during periods of high microcystin concentration in a eutrophic lake were well-adapted to surviving microcystin exposure [167]. Resting eggs formed during periods of low microcystin concentration experienced higher inhibition of growth when exposed to microcystins [167].

### 2.5. Detection of Microcystins 

Detection of microcystins is a challenging problem for monitoring. Historically, researchers have used antibody-based enzyme-linked immunosorbent assays (ELISAs) to study microcystin concentrations in water and in extracted bloom samples. This strategy is sufficient for relatively clean matrices, like water or algal extracts, but its use becomes limited when applied to fish. ELISAs do not differentiate between free microcystin and its conjugates nor between different congeners of microcystins [168]. In addition, ELISA often cross reacts with other non-microcystin metabolites in tissue, leading to overestimation of the actual toxin concentration in these tissues. Thus, many analyses conducted on tissue matrices using ELISA are susceptible to false positive readings for microcystins. In response, many laboratories have turned to using liquid chromatography tandem mass spectrometry for the analysis of microcystins in fish tissues. This method requires a single transition of the molecular ion to a fragment ion, which is used to identify and quantify the analyte of interest. In tissue matrices, there may be molecules with a mass to charge ratio similar or identical to that of the desired analyte. This can lead to overestimation of the true concentration of microcystins in tissues [169]. Cleanup procedures, including C-18 solid phase extraction cartridges, hexane extraction and charcoal solid phase extraction cartridges, have been used to reduce these interferences. Internal standards, such as thiol-LR, can be used to determine microcystin concentrations in fish tissues through LC-MS analysis, but this does not address the issue of interference from the tissue matrix in the single LC-MS signal of the analyte [170]. A solution to this potential overestimation is through the use of liquid chromatography tandem quadrupole mass spectrometry (LC-MS/MS), which requires fragmentation of the analyte of interest in a collision cell. This produces several fragmentation ions, which can be used to increase confidence in the correct identification of a signal as a microcystin. One ion is designated as the quantitation ion, used to determine the concentration of the microcystin in a sample. An additional ion (or more) can be used as a confirmation ion to positively identify a signal as being from a microcystin.

Liquid chromatography-mass spectrometry (LC-MS) and liquid chromatography tandem mass spectrometry (LC-MS/MS) are also much more useful in determining the speciation of microcystins. The validated procedure of the California Department of Fish and Wildlife uses LC-MS/MS to separate six microcystin variants in both tissue and water samples [171], and Schmidt *et al.* (2013) used LC-MS/MS to detect MC-LR in fish tissues using five LC-MS/MS fragmentation ions [95]. A summary of common procedures in the literature for the detection of MC-LR in animal tissues is given in Table 1.

**Table 1:** Extraction and cleanup protocols for microcystin-LR in tissue from the literature.

Reference	Year	Cleanup method	Analysis	Recovery of microcystin-LR
[172]	2005	C18 solid phase extraction	LC-MS	~57% (estimated from Figure 3 of reference [172])
[129]	2005	C18 solid phase extraction	LC-MS and ELISA	68%–96%
[173]	2005	C18 solid phase extraction	LC-MS and ELISA	44%–101%
[174]	2007	-	ELISA	>25%
[105]	2007	Waters Oasis solid phase extraction	LC-Photodiode array	>85%
[175]	2008	Waters Oasis solid phase extraction, Silica gel	LC-MS	>90%
[171]	2009	500 mg C18 solid phase extraction	LC-MS/MS	74%–125%
[170]	2009	-	LC-MS	80%–99%
[114]	2009	-	LC-MS	68%–73%
[95]	2013	Charcoal solid phase extraction	LC-MS/MS	54%–106%

The analysis of microcystins in tissues is further complicated by the large variation in recoveries of MC-LR between methods (Table 1), making it difficult to select a method for monitoring purposes. The fact that MC-LR has been emphasized over other variants also complicates these methods, as MC-LR may not be present in all microcystin-producing blooms. It is possible that other microcystin congeners would be overlooked using these methods, as the recovery of other variants of microcystins may not be identical to that of MC-LR. Other microcystin congeners and their metabolic products possess varying degrees of toxicity by PPIA [29] and by the mouse bioassay [85]. The variants of microcystins present and detected for monitoring will impact the toxicity of tissues and are an important component in monitoring.

## 3. Toxicokinetics

Information on the rate of microcystin metabolism in various organisms is limited, yet is essential for accurate toxicity assessments regarding microcystin exposure due to alterations in the environment, as well as via food web transfer. Potential microcystin transfer between tissues may vary by organism and impact bioaccumulation or biodilution. Speciation, prior exposure to microcystins, age of the organism and size of the organism may affect these processes. Models are useful tools for a better understanding of the toxicokinetics of microcystins in organisms and may help simplify factors for forming guidelines for human exposure.

### 3.1. One-Compartment Models

**Figure 4:**
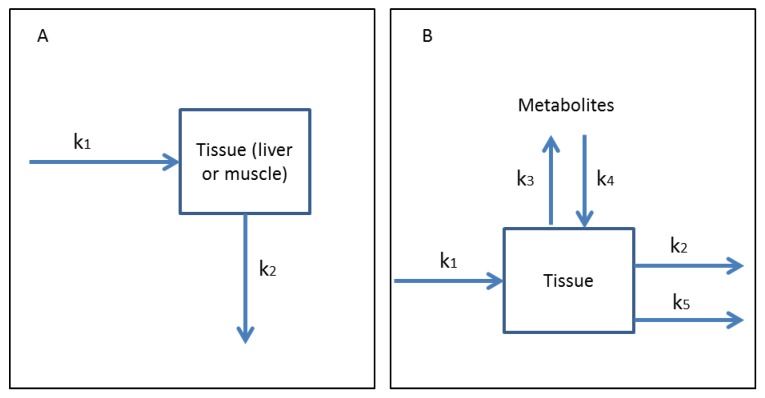
(**A**) One-compartment model for uptake and elimination of a toxin from an organism. Uptake rate *k*_1_ and elimination rate *k*_2_ are used to determine the duration of time a toxin is metabolized and eliminated from the organism during a set duration of exposure. (**B**) One-compartment model for uptake and elimination of a parent toxin and any products from the metabolism of the parent toxin. Kinetic constants *k*_1_ and *k*_2_ represent uptake and elimination of the parent toxin by the tissue, respectively. The rate of the formation of metabolites from the parent toxin (*k*_3_) and uptake of these metabolites into the tissue (*k*_4_) are modeled. The elimination of metabolites from the tissue is represented by the kinetic constant *k*_5_. Uptake and elimination of the parent and metabolized toxin by individual organs are not considered.

A one-compartment model assumes that the concentration of a toxin in the tissue is proportional to the concentration of toxin in the surrounding media. A single compartment (tissue), rather than differentiation by organ, is used to represent uptake and elimination. Uptake of a toxin is described with a first-order uptake constant (*k*_1_) (Figure 4A) [176]. Elimination from tissue is described using an exponential term (*k*_2_) (Figure 4A). This model only considers the parent compound and not metabolic products (GSH conjugates, *etc*.).

When metabolites of a toxin are formed, additional kinetic constants must be considered (Figure 4B). The rate constant for the uptake of the parent toxin into tissues (*k*_1_) is considered along with the rate of the formation of metabolites (*k*_3_) and the uptake of these metabolic products of the parent toxin by the tissue (*k*_4_) (Figure 4B). Rate constants for the elimination of the parent toxin (*k*_2_) and of the metabolites (*k*_5_) are also considered, although different metabolites may not have the same rate constant in an organism (Figure 4B).

Dyble *et al.* (2011) presented a one-compartment, first order kinetic model for MC-LR elimination in yellow perch (Figure 4A) [177]. Although liver and muscle MC-LR concentrations were analyzed, the kinetic model was not differentiated by tissue type [177]. This study used only a single oral dose of MC-LR administered through a food pellet. The maximum MC-LR concentration was obtained in the liver tissue after 10 h and in muscle tissue after 12 h (estimated from Figure 2 of reference [177]). This model may not be applicable to organisms in natural systems with prolonged or variable exposure.

Concentrations in fish liver increased rapidly between 4 and 6 h of exposure and in muscle between 9 and 12 h (estimated from Figure 2 of reference [177]). MC-LR was detectable in the tank water after 4 h and increased throughout the experiment. ELISA was used to measure microcystins; thus, conjugated metabolites, such as GSH derivatives, could not be measured separately [168,177]. Intestinal uptake by fish was not analyzed, but a fish digestion model predicted that 90% of MC-LR administered by food pellet would pass through the gut within 24 h. This model suggested that consumption of contaminated fish tissues was not a major pathway for human exposure to MC-LR [177]. The accumulation and elimination of MC-LR in fish did not appear to be dose dependent. This suggests that the concentration of MC-LR in fish reached a threshold value after which the organism was unable to process the toxin or that prior exposure to the fish resulted in different rates of elimination of MC-LR [177].

The maximum MC-LR concentration in the hepatopancreas of bivalves was observed after five days of oral exposure to MC-LR in a one-compartment model [133]. MC-LR concentration rapidly increased for five days of oral exposure and reached a steady-state concentration, even when bivalves were continuously dosed for 10 days. This implies a maximum threshold MC-LR concentration may have been reached. After bivalves were introduced to non-toxic food for 15 days, rapid elimination of MC-LR was observed [133]. The test organisms used in this study were taken from a lake with prior toxic *Microcystis* blooms and depurated in non-toxic water before the experiment began. This prior exposure of these bivalves to microcystins may have impacted the threshold concentration accumulated in tissues, so as to prevent mortality. The toxicokinetic model here also varied according to time of year in which the bivalves were harvested. Higher concentrations of MC-LR were observed in tissues during colder seasons, as an increase in temperature increased the rate of elimination [133].

### 3.2. Multi-Compartmental Models

**Figure 5:**
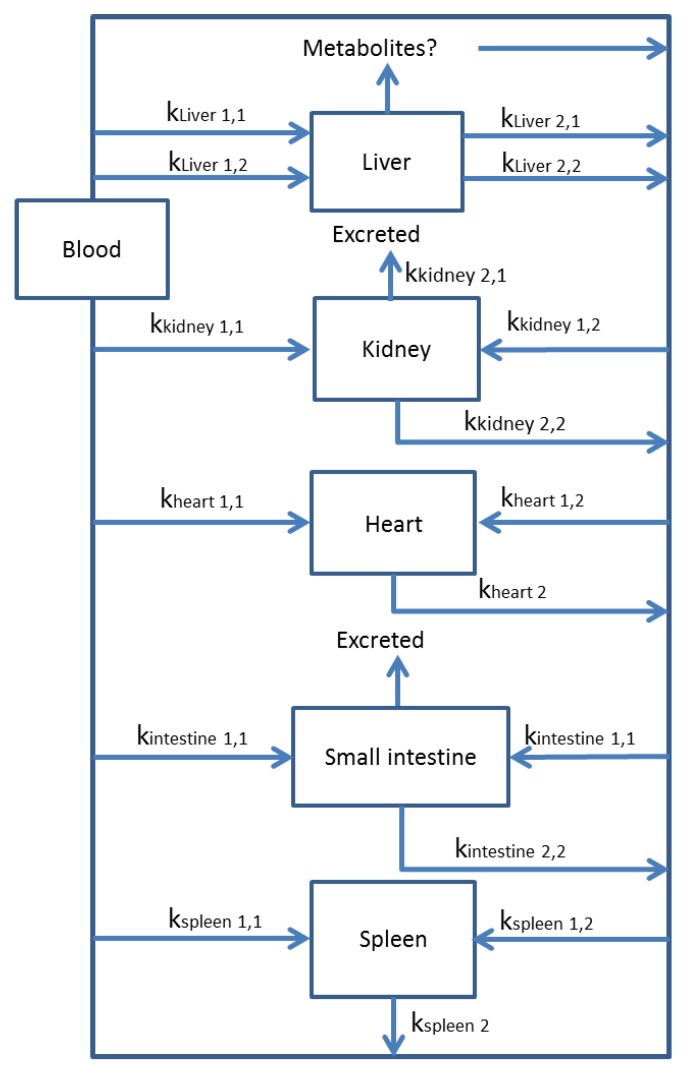
Multi-compartment model for uptake and elimination of a toxin from an organism. Multiple kinetic constants for both uptake (*k*_1_) and elimination (*k*_2_) are possible due to the circulation of metabolite products through the organism and multiple pathways for the toxin to move through. Excretion by different organs also adds additional elimination constants to the model, such as in the kidney (*k*_kidney__2,1_) and small intestine (*k*_intestine__2,1_).

A multi-compartmental model produces multiple kinetic constants. It can also include multiple elimination constants that may be organ-specific. For example, the kidney and small intestine have two possible elimination routes: excretion through the urinary or gastrointestinal tracts or release of non-excreted toxin back into the blood (Figure 5). All organs have the potential to release non-metabolized toxin back into the blood for subsequent uptake by other organs. The liver has the potential to form and release metabolites into the blood or to the bile [178]. Phase II metabolites in bile may be hydrolyzed and reabsorbed by the small intestine in the enterohepatic cycle, reintroducing the toxin to the blood [178]. There are also unique uptake constants for uptake of the parent toxin into each organ (Figure 5). Unique kinetic constants for the elimination of a parent toxin from each specific organ are also assigned to account for differences between organs (Figure 5). Several studies have been conducted on microcystin uptake and elimination using multi-compartmental models.

Beasley and Stotts prepared a multi-compartment model for MC-LR metabolism in pigs. MC-LR concentration in blood peaked after 90 min following intravenous injection [179]. No conjugated metabolites were identified. After 4 h, the highest distribution of the radiolabeled MC-LR was in the liver, followed by kidneys, heart, small intestine and spleen (Figure 5) [179]. The presence of MC-LR in the kidney, small intestine and spleen are indicative of elimination. However, it is not clear at what point within the 4-h window this elimination began. MC-LR concentration was detectable in the intestines of pigs after 4 h of exposure, but the majority of MC-LR was concentrated in the liver [179]. MC-LR was detected in the bile of pigs after only 12 min of exposure, indicating that biliary elimination of MC-LR may be a major elimination pathway in pigs. Smaller doses of MC-LR resulted in higher concentrations in tissues after exposure than larger doses of MC-LR [179]. The metabolism of MC-LR is faster with lower doses than with higher doses in pigs. Higher concentrations of MC-LR could be overwhelming the elimination mechanisms of organisms, as binding of microcystins to protein phosphatases in cells is dose dependent [180]. At higher concentrations, elimination pathways may be unable to keep up with binding of microcystins to protein phosphatases.

The compartmentalization of MC-LR in organs of mice after intraperitoneal injection has also been the subject of several studies. Lin (1994) observed MC-LR in serum and liver after 15 min of exposure, reaching a maximum concentration in serum after 2 h and in liver after 12 h [181]. No additional organs were analyzed. Robinson *et al.* (1990) reported maximum MC-LR concentration in liver, followed by intestine and kidney after 6 h of exposure [182]. Only small concentrations were detected in heart, spleen, lung and muscle. Like the Beasley and Stotts’ (1994) study, intestine and kidney concentrations of MC-LR indicated that these were elimination routes [179]. Brooks *et al*. (1987) observed uptake of 70% of microcystin by the liver after 1 min of exposure, which increased to 90% after 3 h [183]. Uptake into other organs was less than 10%, indicating that the liver is taking up microcystin at a faster rate or containing it more efficiently than other organs in mice.

The concentration of microcystins was higher in the liver than in the muscle of common carp and silver carp after exposure to a bloom of toxic *Microcystis* for two months [174]. Rapid elimination of microcystins from both muscle and liver tissue was observed, with a higher rate of elimination in muscle than in liver. Elimination of microcystins was species-specific, as the half-life of microcystins in silver carp was shorter than that in common carp [174]. Under identical experimental conditions, the half-life of microcystins was only 0.7 days in muscle and 3.5 days in liver of silver carp, as compared to 2.8 days in muscle and 8.4 days in liver of common carp. This evidence suggests that selected species of fish have more efficient mechanisms of microcystin elimination than others.

Interestingly, Lei (2008) reported the highest microcystin concentrations in the blood, heart and kidney in crucian carp administered a mixture of MC-RR and MC-LR intraperitoneally [184]. The distribution of microcystins in this organism via the blood was an important process. The comparatively low concentration of MC-LR in the liver (0.07% of total microcystins) was different from findings in most other studies. The application of a combination of microcystin congeners may result in different distribution of microcystins between organs than observed using MC-LR alone.

Microcystin uptake and distribution within organisms is highly variable. Factors that influence this distribution include the test species, the method in which microcystins were administered to test animals, if the organisms were aquatic or terrestrial, the congener of the microcystin, the temperature of the surrounding water and any prior exposure of organisms to microcystins. The possible re-introduction of microcystins into the blood after absorption in the small intestine could also impact metabolism. The possibility that multiple pathways for the metabolism of microcystins exist in various organisms must also be considered.

### 3.3. Microcystin Conjugate Toxicokinetics

Little is known on how rapidly the different microcystin metabolites are formed. GSH-MC-LR was detected in the macrophyte, *Ceratophyllum demersum*, 24 h after exposure to MC-LR [87,90]. However, the study only analyzed the single conjugate species without further investigation into the rate of metabolism or metabolic pathway. An unidentified product was found by MALDI-TOF-MS with a retention time less than that of free MC-LR, but after MC-LR-GSH in *Daphnia* *magna*, *C. demersum* and the fish *Danio rerio* [87]. This product could be the MC-LR-Cys conjugate, but the characterization by mass spectrometry was not reported. A similar unidentified product was found in the hepatic cytosol of mice after intravenous injection after 1 and 6 h of exposure [96]. After six days of exposure, two additional products were observed by ultraviolet visible spectroscopy [96]. These three peaks could be conjugated microcystins, but were not further identified. 

The MC-LR-Cys conjugate was more prevalent in fish (*Hypophthalmichthys molitrix*) tissue than the MC-LR-GSH species [106]. It was unclear if this represented direct formation of the MC-LR-Cys conjugate from free microcystin or if the conversion from MC-LR-GSH into MC-LR-Cys was a very rapid process. The GSH and Cys conjugates of both microcystin-RR and MC-LR were identified in mouse and rat liver, respectively, after 24 h of exposure [102]. Metabolism of MC-LR followed the same metabolic pathway as MC-RR, through GSH and Cys intermediates [102]. Unfortunately, there was little information on how quickly the metabolism of both the MC-LR and MC-RR species occurred.

The rate of microcystin metabolism differs between species, as does the degree of resistance to microcystin toxicity. Current information on the rate of MC-LR metabolism in different species of test organisms is limited. Toxin concentration in the hepatopancreas of *Unio douglasiae* increased for five days after being fed the MC-LR-producing cyanobacteria, *Microcystis spp.* [133]. After five days, hepatopancreatic MC-LR concentrations leveled off in a steady-state fashion. An increase in GST activity was observed for different organs of *Mytilus galloprovincialis* when exposed to microcystins [136]. GST activity increased when exposed to MC-LR for a 24-h period in *Daphnia magna*, *Ceratophyllum demersum* and *Danio rerio* [87]. The observed increase in GST activity suggests that MC-LR was being transformed to the glutathione conjugate. In contrast, a marked decrease in GST activity in the goldfish, *Carassius auratus* L., occurred after 24 h of MC-LR exposure [140].

Zooplankton also showed differentiation in response to microcystins. The estimated 48-h LD_50_ is between 10.2 and 18.3 ng of MC-LR per 1 mg of *Daphnia* (fresh weight) for *D. galeata* over the course of two days [185]. This information does not tell how prolonged exposure to MC-LR affects *D. galeata*, nor can it be used to determine how quickly the metabolism of MC-LR occurs. The LD_50_ for *E. affinis* is two orders of magnitude smaller than that of *D. magna* [162]. Unknown factors include how the toxin is accumulated within the organism or if there is a depuration period following exposure.

Below this estimated LD_50_ for MC-LR, it is possible that test organisms would metabolize MC-LR rather than suffer mortality. Sadler and von Elert (2014) did not observe any conjugation of MC-LR to GSH during a 24-h exposure of *D. magna* to microcystin [186]. MC-LR was administered to the *Daphnia* using toxic *Microcystis* as a food source for 24 h, followed by transfer to clean media and their feeding on *Cryptomonas spp*. The MC-LR concentration did not change throughout the experiment, and no transformation of the parent toxin was observed. MC-LR was not assimilated by *Daphnia*, as no MC-LR was detected after feeding with *Cryptomonas spp.* [186]. Absorption of microcystin may not have occurred or a detoxification mechanism other than the GSH metabolic pathway may be active in *Daphnia*. The elimination of MC-LR directly into the surrounding media without any biological alteration using a P-glycoprotein was suggested as a possible mechanism. 

It has been suggested that glutathione conjugation to microcystins is a reversible process *in vivo* [187]. MC-RR was detected in tissues of fish injected interperitoneally with MC-RR-GSH and MC-RR-cysteine. Reversal of Michael addition reactions require low pH, suggesting that there may be an enzymatic process liberating MC-RR from its conjugated form *in vivo*. This could have major implications for monitoring, since the conjugates could comprise a large portion of the toxin pool in a natural system [91]. The metabolism of the conjugates by fish could transform microcystins into their original and most toxic form for possible excretion and uptake by other organisms in the environment. Moreover, the presence of MC-RR was dominant in the blood, not in the liver tissues [187]. This suggests another potential compartment for microcystins to occupy in fish and the importance of transport processes between organs. This could also potentially explain the presence of microcystins found in fish muscle tissues rather than liver tissues. More studies on this reversible conjugation are essential for a more complete understanding of the toxicity, transport and transformation of microcystins in living cells.

## 4. Conclusions and Future Direction

Microcystins are prone to physical, biological and chemical alterations in the environment. Detection of microcystins in tissues has been well-documented, yet the species of the organism and high variability between organisms of the same species with regard to uptake of microcystins complicate calculations for safety guidelines. Abiotic and microbial breakdown of microcystins, as well as the potential toxicity of the conjugated MC-LR metabolites produced by organisms in their tissues also makes the determination of human health guidelines difficult. Limited knowledge on the rate of metabolism and rate of formation of the conjugated MC-LR products is available. The potential human health risk of microcystin intoxication due to food web transfer of microcystins also remains uncertain, although microcystins and their metabolite products have been detected in some organisms of different trophic levels. The human health impact of these metabolized products being excreted and released into the environment is also uncertain. 

Additional information on the rate of formation of these conjugates, as well as the rate of degradation of microcystins by microbes is needed to provide a more complete understanding of the role metabolism plays in the toxicity of MC-LR in natural systems. More data on the fate of microcystin conjugates *in vivo* are also needed to better understand the potential risk of human intoxication from tissues. The lack of the GSH-conjugated products in *Daphnia* indicates that other detoxification mechanisms may be dominant over the GSH conjugation pathway and that the use of the GSH pathway for MC-LR metabolism and elimination may not be conserved across the majority of species. 

The number of microcystins and bioactive metabolites produced by cyanobacteria and impacted species is continually increasing as new and more advanced chemical techniques are applied to the identification of these compounds [188]. Microcystin congeners other than MC-LR, as well as any possible metabolites are integral to making accurate toxicity assessments and should be incorporated into the implementation of human health guidelines. One approach is to use cell-based effect assays, such as enzyme-based assays, such as the protein phosphatase inhibition assay. These assays may be effective alternatives to current monitoring techniques, especially for water samples where the concentration of matrix interferences is low. However, care must be taken to ensure that the response in these *in vitro* assays is representative of the *in vivo* toxicity, as properties, such as uptake and transport, are rarely captured by these *in vitro* tests. Structure-based assays, such as ELISA, may also give an integrated microcystin value in a sample, but it is often difficult to convert this value into a toxic effect without the knowledge of the individual congeners present. A better understanding of the occurrence of different congeners, their metabolic products and the toxicity of the between different microcystin congeners would help in this regard. 

The analysis of tissue samples require different approaches, as interferences from the tissue matrix can result in false positives in both effect-based assays, such as PPIA, or structure-based assays, such as ELISA [114]. Improved cleanup methods may be able to remove these interfering compounds from the tissue matrix. Alternatively, new effect-based assays based on the mode of action of microcystins in cells may be identified that may be less sensitive to these matrix affects. Such assays would be beneficial as a screening tool for the presence of microcystins in tissues. 

A second approach is to use an analysis for individual microcystin compounds. MALDI-TOF, LC-MS and LC-MS/MS are examples of such an approach, which has been applied to water and tissue samples. Analyses focus on individual compounds. The advantage of an analysis is that it is able to distinguish between different congeners of microcystins in the sample. The disadvantage is that the analytical method only considers those toxins that the operator asks it to consider, such as with LC-MS/MS, where a quantitation and confirmation ion are obtained for a particular compound of interest. With an ever-increasing number of microcystins and their metabolites being identified, these analytical methods would need to be updated constantly to incorporate new toxins. In addition, there are many compounds in complex matrices whose mass to charge ratios are similar to that of the desired analyte. Thus, both a quantitation and, possibly, multiple confirmation ions for each congener, metabolite or compound of interest would be needed. Improvements in sample cleanup techniques, as well as a better understanding of the distribution of the different congeners and metabolites in nature are essential to guide the operator in the selection of the correct method.

In practice, the ideal approach to monitoring of microcystins in tissues is likely to include a combination of assays and analyses. An effect-based assay could be used first as a screening tool to rapidly eliminate those samples that do not contain any toxins. This initial screening could then be followed up by more specific analysis using LC-MS(/MS) to confirm that the toxin(s) in question are indeed present, to eliminate potential false positives and to identify the presence of new or unknown toxins in the sample.
